# Pilot Study on Using Large Language Models for Educational Resource Development in Japanese Radiological Technologist Exams

**DOI:** 10.1007/s40670-024-02251-1

**Published:** 2025-01-18

**Authors:** Tatsuya Kondo, Masashi Okamoto, Yohan Kondo

**Affiliations:** Department of Radiological Technology, Graduate School of Health Sciences, Niigata University, 2-746 Asahimachi-dori, Chuo-ku, Niigata, 951-8518 Japan

**Keywords:** Large Language Models, Radiological Technologist Training, Education Technology, Artificial Intelligence in Education, Learning Enhancement

## Abstract

In this study, we explored the potential application of large language models (LLMs) to the development of educational resources for medical licensure exams in non-English-speaking contexts, focusing on the Japanese Radiological Technologist National Exam. We categorized multiple-choice questions into image-based, calculation, and textual types. We generated explanatory texts using Copilot, an LLM integrated with Microsoft Bing, and assessed their quality on a 0–4-point scale. LLMs achieved high performance for textual questions, which demonstrated their strong capability to process specialized content. However, we identified challenges in generating accurate formulas and performing calculations for calculation questions, as well as in interpreting complex medical images in image-based questions. To address these issues, we suggest using LLMs with programming functionalities for calculations and using keyword-based prompts for medical image interpretation. The findings highlight the active role of educators in managing LLM-supported learning environments, particularly by validating outputs and providing supplementary guidance to ensure accuracy. Furthermore, the rapid evolution of LLM technology necessitates continuous adaptation of utilization strategies to align with their advancing capabilities. In this study, we underscored the potential of LLMs to enhance educational practices in non-English-speaking regions, while addressing critical challenges to improve their reliability and utility.

## Introduction

In medical education, the use of multiple-choice questions (MCQs) is an essential tool for learning and evaluation; however, it is challenging to use them effectively [1–4]. MCQs are widely used in healthcare professional licensure exams across many countries, including Japan, and have proven to be valuable in assessing levels of achievement and knowledge. However, the constant need for new questions and the error-prone nature of MCQ creation present significant obstacles [3]. Approaches such as ontology-based automated generation exist; however, they often lead to monotonous questions, which highlights the need for more dynamic solutions [1, 5]. Additionally, research that focuses on question creation and solving skills is prevalent, but there is a notable gap in exploring the development of explanatory materials for MCQs. These materials include explanatory texts that clarify the questions and answer choices, in addition to diagrams, graphs, and supplementary content.

Large language models (LLMs) are a promising technological application in medical education [4, 6–8]. LLMs are advanced artificial intelligence systems designed to process and generate human-like text based on vast amounts of training data. These models can perform tasks such as language translation, sentiment analysis, image captioning, text summarization, question-answering systems, content moderation, text paraphrasing, text completion and prediction, programming code generation, and debugging, and are a versatile tool for diverse interactive applications [8]. LLM development has introduced multimodal capabilities that allow these models to process data formats other than text, such as images. This advancement has led to the application of LLMs to medical imaging research [9]. LLMs have demonstrated the capacity to pass medical exams in English [10–12] and have achieved success in various Japanese medical licensing exams, including physician, dental, pharmaceutical, and nursing exams, in addition to specialized radiology exams [13–16]. Applying LLMs to medical education offers various benefits, such as personalized learning experiences, key point summaries, generation of practice questions, access to a vast amount of information, reference to resources, assistance in reviewing MCQs, and multilingual medical education. For educators and instructors, LLMs are expected to provide approaches to enhance student engagement [4, 6]. In previous studies on LLMs in medical education, researchers have explored their utility as training tools for healthcare professionals [17, 18], the generation of clinical vignettes [19], and medical writing assistance [20].

Several concerns have been raised about the application of LLMs to medical education, as noted in various studies [4, 6–8]. These include the risk of promoting academic dishonesty, over-reliance on artificial intelligence (AI), the potential dilution of critical thinking skills, and the accuracy and reliability of content generated by LLMs [6]. In the context of MCQs, the accuracy of LLMs remains a concern [4, 21]. It is crucial to perform thorough evaluations and tests for erroneous responses or “hallucinations” (i.e., generation of incorrect or fabricated information not found in the training data). Educators must emphasize that LLMs and related applications cannot replace comprehensive lectures or textbooks and may provide inaccurate information based on inadequate user prompts [6]. Addressing these concerns is vital for applying LLMs to medical education.

In this study, we aim to create and evaluate educational resources using LLMs for medical MCQs in Japan, that is, a non-English-speaking region. Focusing on radiological technologist exams as a case study, we envision scenarios in which educators at radiology technologist training schools use LLMs to develop explanatory materials for medical qualification exams. Four key reasons support this scenario: (1) using LLMs can mitigate concerns about their accuracy when used by educators, (2) it uncovers potential concerns when students independently use LLMs for learning, (3) targeting MCQs for limited-participant medical qualifications in non-English-speaking regions has broad applications, and (4) radiological technology actively applies AI, with the possibility to examine medical imaging questions. This sector relies heavily on medical imaging and anticipates the application of multimodal functionalities [22]. In this study, we assess the quality of explanatory texts generated by LLMs for past national exam questions and explore considerations and methodologies for using LLMs in learning support.

## Methods

In this study, we used LLMs to generate explanatory texts for MCQs, focusing on their quality evaluation. Table 1 provides a concise overview of the study, detailing the duration of LLM use, the specific LLM service used, the nature of the MCQs examined, their classification method, and the approach to assessing the explanatory texts.

**Table 1:** Summary of the search strategy

Items	Specification
Duration using LLMs^a^	February 28, 2024, to March 21, 2024
LLMs^a^ service used	Microsoft Bing Copilot
MCQs^b^ examined	Morning session of the Radiological Technologist National Exam conducted in Japan, February 2022
Number of MCQs^b^	100
Excluded MCQs^b^	None
Modified MCQs^b^	3
Category of MCQs^b^	Image-based, calculation, textual
Method for evaluating explanatory texts	Four criteria:
	(1) Generation of a bug-free explanatory text in a single input/output cycle;
	(2) Correct explanatory text of the correct answer choice;
	(3) Correct explanatory texts of incorrect answer choices;
	(4) Accurate explanatory text of specialized terminology used in the question

^a^*LLMs*, large language models; ^b^*MCQs*, multiple-choice questions

### Targeted National Exam Questions

In this study, we modified and used a subset of 100 MCQs from the 2022 Radiological Technologist National Exam in Japan. This exam is conducted annually and is divided into two sessions (morning and afternoon) on the same day. In the February 2022 exam, 100 MCQs were administered in each session and candidates were required to complete both sessions. The subjects covered in the morning and afternoon sessions were identical. The exam questions are publicly available on the official website of the Japanese Ministry of Health, Labour and Welfare [23]. For this study, we focused on 100 MCQs from the morning session and categorized them into three types: image-based, calculation, and text-based questions.

#### Correction of the Question Statement

For questions in the examination where a correct option was not initially present, we adjusted some choices to include a valid, correct answer. This modification was essential to ensure that these questions were appropriate and usable for our study.

#### Classification of Exam Questions

We categorized the exam questions on the basis of the questioning format rather than the subject matter or topic area. Image-based questions were defined as those using medical images, photographs, circuit diagrams, or dimension drawings. Calculation questions were defined as those that did not fall into the image-based category and had all choices in numerical form. Textual questions, distinct from image-based and calculation questions, were defined as purely text-based questions that assessed fundamental knowledge or thought processes. The distribution of questions was as follows: 19 image-based, nine calculation, and 72 textual questions.

### Method Used to Create Explanatory Texts

We generated explanatory texts using Copilot integrated into Microsoft Bing, which is a type of LLM that applies natural language processing. Copilot generates text based on provided prompts and has the capability to perform internet searches, using web-based information in its responses. It also supports image input, thereby allowing the use of both images and text. All prompts input into Copilot were written in Japanese. Table 2 provides a specific example of a prompt translated from Japanese to English. For image-based questions, we used image data obtained in PDF format. We set the PDF display to 200% magnification and captured screenshots of the images, which we then input into Copilot. We exported and saved the explanatory texts generated by Copilot from these images in PDF format. It should be noted that we did not retain information from referenced websites when exporting these texts in PDF format.

**Table 2:** Example of a prompt translated from Japanese to English, with adjustments made within [] to suit each question

Example of a typed prompt
[A001]-Question Text:
[Which of the following pairs of element symbols and names is correct?
1. Cu—Chromium
2. Ge—Gallium
3. Ce—Selenium
4. Lu—Lutetium
5. Ta—Thallium]
Please create detailed explanatory text for this question to assist radiological technologist students in their studies. Export the answer for use in learning. The format for the response should be as follows:
[A001]-Question Text:
Answer Number:
Explanatory Text:
Response to the example prompt
A001-Question Text: Which of the following pairs of element symbols and names is correct?
1. Cu—Chromium
2. Ge—Gallium
3. Ce—Selenium
4. Lu—Lutetium
5. Ta—Thallium
Explanatory Text: This question asks you to choose the correct combination of element symbol and element name. The correct combination among the choices is 4. Lu—Lutetium
1. Cu is the element symbol for Copper and Cr is the element symbol for Chromium. 2. Ge is the element symbol for Germanium, and Ga is the element symbol for Gallium. 3. Ce is the element symbol for cerium and Se is the element symbol for selenium. 4. Lu is the element symbol for lutetium, which is the correct answer. 5. Ta is the element symbol for tantalum and Tl is the element symbol for thallium
Therefore, the correct answer is 4. Lu—Lutetium. These questions are designed to check your knowledge of the elements and to measure your understanding of the basic science required of a radiologist. Accurate memorization of the symbols and names of the elements is important in the professional fields of radiological technologists, such as radiation physics and radiobiology

### Method for Evaluating Explanatory Texts

The evaluation of the explanatory texts involved both quantitative and qualitative methods. We conducted a quality evaluation of the explanatory texts by assessing key aspects, such as the tool’s usability and stability, the provision of the rationale for correct and incorrect answers in MCQs, and the level of specialized knowledge in the medical field. The quantitative assessment involved a cumulative scoring system and a tally of the number of questions containing errors. Additionally, two instructors conducted qualitative evaluations in which they reviewed the explanatory texts for their educational adequacy and accuracy.

#### Quantitative Evaluation of Explanatory Texts

We conducted a quantitative evaluation of the explanatory texts generated by LLMs to assess their quality using four specific criteria: (1) whether the explanatory text was generated in a single interaction without technical errors, (2) providing a correct explanatory text for the correct answer choice, (3) offering correct explanatory texts for incorrect answer choices, and (4) accurately explaining specialized terminology used in the question. Two qualified radiological technologist instructors scored each explanatory text independently and assigned 1 point for meeting each criterion, which resulted in a total score ranging from 0 to 4 points per question.

Criterion 1 assessed the technical integrity of the explanatory text generation process, ensuring that outputs were free from issues, such as repeated phrases, incomplete responses, or errors during generation. This criterion focused on the coherence and completeness of the process rather than content accuracy. Criterion 2 evaluated the correctness of explanatory texts for the correct answer choice, ensuring logical alignment with the question and, for calculation questions, adherence to correct formulas and computations. Criterion 3 examined the accuracy of explanatory texts for incorrect answer choices, verifying clear and valid reasoning for their incorrectness. For calculation questions, this included ensuring accurate computations that guided the correct choice. Criterion 4 focused on the accurate use and explanatory text of specialized terminology, which reflected domain-specific knowledge.

We calculated the average scores for each question category (textual, calculation, and image-based), along with their 95% confidence intervals (CIs) using the *t*-distribution. Additionally, we analyzed the generated texts for recurring errors and recorded the number of questions with inaccuracies to identify areas for improvement.

#### Qualitative Evaluation of Explanatory Texts

Two radiological technologist instructors with national qualifications evaluated the generated explanatory texts qualitatively. This qualitative analysis was agreed on through joint consultation and focused on identifying errors and inaccuracies across different categories. It also involved examining the characteristics of texts that received lower scores in the quantitative evaluation to understand specific issues or recurring patterns in the texts generated by the LLMs. Multiple responses were obtained using the same prompts when necessary to examine the characteristics of the explanatory texts.

## Results

### Quantitative Evaluation

Table 3 displays the number of questions per category, the scores for the explanatory texts, and the 95% CIs. We assessed the quality of the explanatory texts in each category on a 0–4-point scale, where the higher the score, the higher the quality. The average scores indicated that textual questions had the best evaluation, at 3.2 points, whereas image-based questions had the lowest, at 1.7 points.

**Table 3:** Quantitative evaluation of explanatory text quality: average scores of explanatory texts evaluated for each category and their 95% CIs using the *t*-distribution

Category	Number of questions	Scores	
		Average	95% CI^a^
Image-based	19	1.7	[1.02, 2.35]
Calculation	9	3.0	[1.98, 4.02]
Textual	72	3.2	[2.98, 3.44]

^a^*CI*, confidence interval

**Fig. 1:**
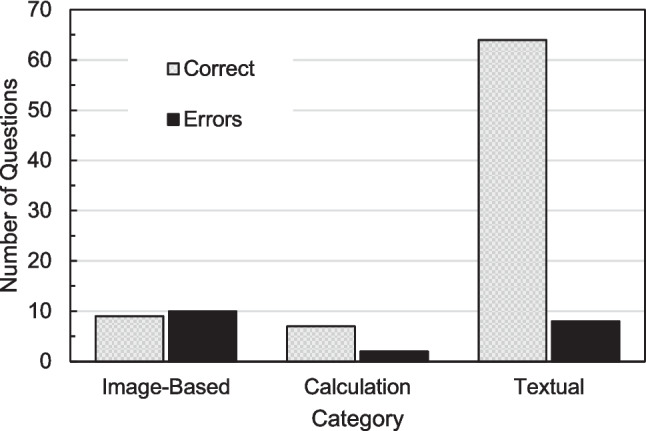
Comparison of the number of questions within each category that resulted in explanatory texts that did not contain errors with the number of questions that resulted in explanatory texts that did contain errors

Figure 1 compares the number of questions within each category that resulted in explanatory texts that did not contain errors with the number of questions that resulted in explanatory texts that did contain errors. Every category had instances of errors in explanatory texts. The proportion of texts with errors was 52.6% for image-based questions, 22.2% for calculation questions, and 11.1% for textual questions.

### Qualitative Evaluation

#### Image-Based Questions

Technical challenges arose in generating explanatory texts from image-based questions. Issues included failure to initiate or complete text generation after image inputs. The system struggled with accurate image recognition and its ability to create corresponding texts.

The system’s explanatory texts for image-based questions exhibited both valuable interpretations and significant inaccuracies. Some instances demonstrated the system’s ability to provide meaningful explanatory texts, even when its image recognition capabilities were limited, as illustrated in Table 4. However, other cases highlighted critical errors, such as the example in Table 5, where the generated text included inaccurate interpretations of visual content. The findings indicate significant challenges in the system’s ability to interpret and explain visual data.

**Table 4:** Example of valuable explanatory text and a qualitative evaluation for an image-based question. The explanatory text was translated from Japanese to English

Response
A021-Question Text: A separate abdominal magnetic resonance (MR) image No. 43 is shown. Which of the following reduces the artifact indicated by the arrow? 1. Extension of TE 2. Extension of TR 3. Combination with a respiratory synchronization method 4. Addition of CHESS pulse 5. Addition of spatial saturation pulses outside the slice
Answer Number: 5
Explanatory Text: This question asks about the method used to reduce the artifact indicated by the arrow in the abdominal MR image. The correct answer is “5. Addition of spatial saturation pulses outside the slice.” This technique, used to improve MR image quality, saturates specific extraneous slice areas to decrease artifacts. It enhances the quality of the captured image, allowing for more accurate diagnoses. Specifically, spatial saturation pulses outside the slice saturate (i.e., nullify the signal of) the regions outside the imaging plane, thereby eliminating unwanted signals from those areas. This can reduce artifacts that appear in the images. The technique is beneficial when imaging organs in motion, such as the abdomen, and suppresses artifacts caused by physiological signals, such as breathing and heartbeats. Therefore, the correct answer to this question is “5. Addition of spatial saturation pulses outside the slice,” which can reduce artifacts in abdominal MR images. This knowledge is crucial for deepening the expertise of radiological technologists and providing higher-quality images. The above is a detailed explanatory text of the question. We hope it will benefit the students of radiological technologist training schools
Qualitative Evaluation
The image in an MR scan targeted the liver, showing the heart and liver on the same slice in a fat-suppressed T_1_-weighted axial image. The arrow on the image indicated an artifact, which was identified as either a ghost artifact or motion artifact occurring around the aorta. The explanatory text did not explicitly mention the type of artifact in the image. However, from the content associated with answer number 5, it inferred the type of artifact that could be suppressed and explained under what circumstances suppression would be effective. Therefore, although we concluded that the image was not recognized, we deemed valid explanatory text to have been presented

**Table 5:** Example of explanatory text and a qualitative evaluation for an image-based question containing errors. The explanatory text was translated from Japanese to English. The alphabetic characters A–E in the example correspond to the first five syllables of the Japanese katakana alphabet

Response
A015-Question Text: An MRCP image is provided separately. Which of the following is the main pancreatic duct?
1. A 2. B 3. C 4. D 5. E
Answer Number: 4
Explanatory Text: In this MRCP image, several structures are labeled. The main pancreatic duct, indicated by answer number 4, is the widest and centrally located duct compared with other structures in the image. Understanding the position and shape of the main pancreatic duct is crucial for radiological technologists because it plays a vital role in diagnosing liver and gallbladder diseases. Accurate identification of this and other biliary structures is essential because abnormalities can lead to symptoms such as jaundice, cirrhosis, or cholelithiasis. Remembering the main pancreatic duct as “D” can aid future patient diagnoses and treatment plans. Use this knowledge to enhance your skills as a radiological technologist and prepare for the national exam. Good luck!
Details of the attached images
The question presented an MRCP image targeting the pancreatic-hepatobiliary system. In the image, five arrows were depicted, each pointing toward an organ and labeled with symbols for differentiation. The arrows corresponded to the following organs: common hepatic duct, gallbladder, common bile duct, main pancreatic duct, and renal pelvis. The symbols used for arrow identification mirrored the first five characters of the Japanese katakana alphabet, similar to the choice options. The image can be downloaded from the official website of the Japanese Ministry of Health, Labour and Welfare [23]
Qualitative Evaluation
The correct organ-arrow combination pertained to the main pancreatic duct, which is the primary route for transporting pancreatic juice to the duodenum. However, the explanatory text incorrectly stated that the main pancreatic duct was the primary pathway for bile flow from the liver to the duodenum. Additionally, despite the common bile duct being the thickest structure in the image, the text erroneously described the main pancreatic duct as the thickest structure. This led us to conclude that the attached image was not accurately recognized in the explanatory text

#### Calculation Questions

There were instances of inaccuracies in the output formulas and errors in calculations. An example of explanatory text for a calculation question containing errors is presented in Table 6, originally composed in Japanese and then translated into English.

**Table 6:** Example of explanatory text and a qualitative evaluation for a calculation question containing errors. The explanatory text was translated from Japanese to English

Response
A093- Question Text: In an angiographic examination, an X-ray tube with a focal spot size of 0.3 mm was used, and radiography was performed with a distance of 100 cm between the focal spot and the image receptor. When the vessel to be contrasted is 20 cm from the image receptor at the focal spot, which of the following is closest to the size [μm] of this vessel’s geometric irregularity (penumbra)?
1. 15
2. 25
3. 35
4. 50
5. 75
Answer Number: 5
Explanatory Text: This question concerns the calculation of geometric insensitivity (penumbra) in radiography. Geometric insensitivity is determined by the size and position of the X-ray source (focal spot), the object’s position, and the image receptor’s position (X-ray film or digital sensor). The size of the penumbra can be calculated using the following formula:
$$\text{size of the penumbra}=\frac{\text{focal spot}\times (\text{distance from the receiver to the object})}{\text{distance from the focus to the receiver}}.$$
In this question, the focal dimension is 0.3 mm, the object’s distance from the receiver is 20 cm, and the distance from the focal point to the receiver is 100 cm. Substituting these values into the above formula, we obtain
$$\text{size of the penumbra}=\frac{0.3\times 20}{100}=0.06\text{ mm}=60\;\mu \text{m}$$
Therefore, the closest to 60 µm among the choices is 75 µm, and the answer number is 5. Thus, in radiography, various parameters must be adjusted to obtain optimal image quality, considering the focal plane dimensions and the position of the object. This question illustrates one example. As a radiologist, you must understand and apply these principles appropriately in a clinical setting
Qualitative Evaluation
An error was identified in the formula. The correct formula is
$$\text{size of the penumbra}=\frac{\text{focal spot size}\times (\text{distance from the receiver to the object})}{\text{distance from the object to the receiver}}.$$
Despite using an incorrect formula, the numerical calculation was performed accurately, yielding the result of 60 µm. The closest correct answer was option 4 (50), but the system incorrectly identified the predefined answer, option 5 (75), as correct

Ten responses were collected using the same prompt to evaluate the reproducibility of formulas generated by LLMs for the calculation question in Table 6. The formulas varied in their combinations of distances involving the focus, object, and receiver, with some responses correctly pairing these distances. The numerical substitution processes fell into two distinct patterns: “0.3 × 1000/200 = 1.5 mm = 1500 µm” and “0.3 × 20/100 = 0.06 mm = 60 µm.”

#### Textual Questions

Frequently, there were no explanatory texts for specialized terminology in the question text or for incorrect answer choices. We also observed instances of inadequately elaborated explanatory texts. Table 7 shows an example of inappropriate explanatory text. The study’s explanatory text mainly focused on explaining artifacts but did not sufficiently cover the topic of accuracy management in medical image monitors. Considering the question’s emphasis on this area, the explanatory text was evaluated as unsuitable because it did not align with the question’s primary intent.

**Table 7:** Example of inappropriate explanatory text and a qualitative evaluation for a textual question translated from Japanese to English

Response
A046-Question Text: In the accuracy management of medical image monitors, which of the following is a visual test item? 1. Artifacts, 2. Luminance Ratio, 3. Contrast Response, 4. Maximum Brightness, 5. Chromaticity
Answer Number: 1
Explanatory text: This question focuses on the visual test items in the medical image monitor’s accuracy management. The correct choice is identified as “Artifacts”. Artifacts refer to unnecessary information or noise not originally present in images, which can arise during image acquisition, processing, or display. Assessing the extent of the artifacts’ influence on images is crucial for accurately managing medical image monitors because artifacts potentially affect diagnoses. Therefore, checking for artifacts in visual test items is essential for maintaining image quality and supporting accurate diagnostics. Remembering that artifacts are the correct answer to this question will aid radiological technologists in making future diagnostic and treatment decisions. This knowledge is vital for enabling radiological technologists to deepen their expertise and provide higher-quality imaging. This detailed explanatory text can benefit students in radiological technologist training schools
Qualitative Evaluation
The explanatory text primarily focused on artifacts and did not sufficiently cover the topic of quality control for medical imaging monitors. The expected information pertained to the quality control of monitors. For example, quality control involves two methods: quantitative testing using measuring instruments and visual inspection. Quantitative testing measures parameters, such as the luminance ratio, contrast response, maximum brightness, and chromaticity. Additionally, a brief explanatory text of each of these testing items is expected to clarify their purpose and relevance

## Discussion

In this study, we evaluated the quality of explanatory texts generated by LLMs for MCQs from past questions from the Japanese Radiological Technologist National Exam, that is, a non-English-speaking context. The results showed that the explanatory texts for many textual questions were of adequate quality, but those for image-based, calculation, and some textual questions were inadequate. This highlights a need for novel approaches when applying LLMs to image-based and calculation questions. Additionally, we identified issues with accuracy in some textual questions, which indicates areas for further improvement in LLM applications in medical education.

The explanatory texts for image-based questions highlighted several challenges specific to interpreting medical images. For image-based questions, as shown in Table 3, the average score was 1.7 out of a maximum of 4 points. Additionally, as illustrated in Fig. 1, 52.6% of the explanatory texts contained errors. Key difficulties included recognizing visual cues, such as arrows and labels pointing to specific features, and accurately interpreting text or numbers embedded within images. Unlike general images used for training, medical images often include specialized elements, such as diagnostic annotations and geometric indicators, which require a nuanced understanding. These limitations make LLMs less reliable for image-based questions in their current form.

When creating explanatory texts for image-based questions, a promising strategy involves using keywords from the question text rather than inputting images directly. Images used in national exams are often too complex for LLMs to interpret accurately because of their specialized medical content. However, these exams frequently test fundamental concepts in medical technology, which can often be addressed through text-based prompts. By focusing on keywords that highlight key features or concepts, LLMs can generate explanatory texts that help students to deduce answers by consulting the corresponding images. For example, questions related to basic anatomical structures, image artifacts, or disease concepts are particularly suited to this approach. As shown in Table 4, accurate explanatory texts aligned with question content can guide effective learning. Furthermore, combining keyword-based prompts with internet image searches allows students to supplement their understanding, making LLMs a useful adjunct in image-focused education.

The explanatory texts for calculation questions highlighted significant challenges in LLMs’ ability to handle numerical accuracy. Issues included incorrect formulas and erroneous arithmetic processes. For example, in the question detailed in Table 6, repeated prompts resulted in varied formulas, incorrectly combining distances between the receptor, object, and focus. The correct formula, which required implicit reasoning about the focal point and object distances, was often misrepresented. This variability reflects the inherent characteristics of LLMs, where responses can differ based on subtle variations in prompts. Moreover, the study reaffirmed the well-documented difficulty of LLMs with basic arithmetic. These findings suggest that integrating LLMs with programming capabilities, such as Python, may enhance their utility for calculation-based tasks. However, a reliance on paid models, such as OpenAI’s ChatGPT-4, raises concerns about equitable access in educational settings.

The explanatory texts for textual questions, which comprised nearly 90% without errors and achieved high average scores, demonstrated sufficient quality. These findings are presented in Table 3 and Fig. 1. This outcome indicates that the explanatory texts for textual questions were of a notably high standard. This aligns with LLMs’ proficiency in text-based information processing and handling specialized terms, which is a trend consistent with prior research [8, 11]. These reports suggest that LLMs can adeptly manage basic specialized terminology.

In this study, we demonstrated a specific method for applying LLMs to medical education. In previous studies, researchers reported on the capabilities of LLMs in the medical field [10–16], their usefulness as training tools [17, 18], and concerns regarding their use in medical education [4, 6–8]. By contrast, we introduced a new application scenario in which educators in radiological technologist training programs use LLMs to develop explanatory materials for medical licensure exams. By focusing on MCQs for radiological technologists in Japan, we addressed challenges associated with a specific and limited examinee population and limited online resources, compounded by the linguistic and contextual constraints of conducting research in Japanese. Despite these challenges, the results demonstrated that LLMs can generate high-quality explanatory texts for specialized content in non-English educational contexts.

The results of this study indicate that educators at training institutions can use current LLMs to generate explanatory texts for textual questions in multiple-choice exams. Using LLMs, educators can mitigate the impact of hallucinations often associated with LLM outputs, enabling the creation of high-quality explanatory texts for MCQ with greater ease. The expected benefits of LLM integration include the ability to address new questions more efficiently, provide personalized learning experiences for individual students, move beyond ontology-based repetitive question patterns, and create multilingual explanatory texts. Furthermore, it became evident that handling medical images requires carefully designed prompts, whereas addressing calculation questions necessitates selecting LLMs capable of using programming languages.

In learning environments where students use LLMs, educators must actively engage with and manage the learning framework. As shown in Fig. 1, prompts composed solely of simple question–answer pairs led to errors across explanatory texts in all categories. Table 7 further illustrates examples of insufficient explanatory texts, highlighting a key challenge in applying LLMs to educational settings [4, 6]. Recognizing inaccuracies in LLM outputs is particularly challenging for beginners in specialized fields. Therefore, educators’ active involvement is essential when students rely on LLMs for learning. Strategies such as encouraging the use of diverse learning resources to reduce dependence on LLMs, facilitating discussions among students or between students and instructors, and leveraging interactive features of LLMs, such as chat-based functionalities, could further enhance learning. These strategies offer promising approaches to effectively address the challenges associated with LLMs in educational contexts.

The limitations of this study include the small number of questions analyzed, restricting the scope to radiological technologists, inadequate exploration of prompts and explanatory text evaluation methods, and limited examination of other LLMs. The reliance on a free LLM raises concerns about long-term availability, rapid performance changes, and capability disparities compared with paid models. Additionally, we based the evaluation methods on independently established criteria without conducting a pilot study to test their validity and reliability. The limited number of evaluators, absence of student verification, and reliance on Japanese-language prompts may further restrict the generalizability of the findings. As shown in Table 6, variations in responses to the same prompt indicate that a replication of this study might yield slightly different results.

## Conclusion

In this study, we demonstrated the potential application of LLMs to medical education in non-English-speaking contexts, particularly MCQs outside the field of medicine. Notably, we highlighted the high-quality generation of explanatory texts for textual questions, thereby indicating LLMs’ capability to process specialized content effectively.

Future challenges include addressing issues with calculation questions and medical images, developing learning environments that incorporate LLMs, and refining evaluation standards. For calculation questions, selecting LLMs capable of handling programming functionalities is a promising approach. For interpreting medical images, alternative strategies, such as keyword-based prompt design, are necessary. Additionally, enhancing the reliability and validity of evaluation criteria requires further refinement. The active involvement of educators in students’ learning environments, complementing LLM-generated outputs, will also be essential for improving the accuracy and effectiveness of education.

LLMs are undergoing rapid technological advancements, leading to significant changes in their performance and accessibility over short periods. It is crucial to adapt to these rapid developments and align LLM utilization strategies with their evolving capabilities. By doing so, LLMs can further unlock their potential to meet diverse educational needs across medical fields in non-English-speaking regions.

## Data Availability

The questions from the national certification exam for Japanese radiological technologists used for the analysis were obtained from the website of the Ministry of Health, Labour and Welfare of Japan. The responses are available from the corresponding author upon reasonable request.
